# Imaging of Alkaline Phosphatase Activity in Bone Tissue

**DOI:** 10.1371/journal.pone.0022608

**Published:** 2011-07-25

**Authors:** Terence P. Gade, Matthew W. Motley, Bradley J. Beattie, Roshni Bhakta, Adele L. Boskey, Jason A. Koutcher, Philipp Mayer-Kuckuk

**Affiliations:** 1 Bone Cell Biology and Imaging Laboratory, Hospital for Special Surgery, New York, New York, United States of America; 2 Mineralized Tissue Laboratory, Hospital for Special Surgery, New York, New York, United States of America; 3 Department of Medical Physics, Memorial Sloan-Kettering Cancer Center, New York, New York, United States of America; Ohio State University, United States of America

## Abstract

The purpose of this study was to develop a paradigm for quantitative molecular imaging of bone cell activity. We hypothesized the feasibility of non-invasive imaging of the osteoblast enzyme alkaline phosphatase (ALP) using a small imaging molecule in combination with ^19^Flourine magnetic resonance spectroscopic imaging (^19^FMRSI). 6, 8-difluoro-4-methylumbelliferyl phosphate (DiFMUP), a fluorinated ALP substrate that is activatable to a fluorescent hydrolysis product was utilized as a prototype small imaging molecule. The molecular structure of DiFMUP includes two Fluorine atoms adjacent to a phosphate group allowing it and its hydrolysis product to be distinguished using ^19^Fluorine magnetic resonance spectroscopy (^19^FMRS) and ^19^FMRSI. ALP-mediated hydrolysis of DiFMUP was tested on osteoblastic cells and bone tissue, using serial measurements of fluorescence activity. Extracellular activation of DiFMUP on ALP-positive mouse bone precursor cells was observed. Concurringly, DiFMUP was also activated on bone derived from rat tibia. Marked inhibition of the cell and tissue activation of DiFMUP was detected after the addition of the ALP inhibitor levamisole. ^19^FMRS and ^19^FMRSI were applied for the non-invasive measurement of DiFMUP hydrolysis. ^19^FMRS revealed a two-peak spectrum representing DiFMUP with an associated chemical shift for the hydrolysis product. Activation of DiFMUP by ALP yielded a characteristic pharmacokinetic profile, which was quantifiable using non-localized ^19^FMRS and enabled the development of a pharmacokinetic model of ALP activity. Application of ^19^FMRSI facilitated anatomically accurate, non-invasive imaging of ALP concentration and activity in rat bone. Thus, ^19^FMRSI represents a promising approach for the quantitative imaging of bone cell activity during bone formation with potential for both preclinical and clinical applications.

## Introduction

Imaging is one of the most important diagnostic tools in the clinical evaluation of patients with musculoskeletal conditions [1], [2]. The majority of current clinical musculoskeletal imaging strategies are designed to detect abnormalities in bone morphology as indicators of disease. For example, radiographs and computed tomography measure tissue mineral density to visualize bone anatomy. Similarly, current MR imaging techniques measure proton (^1^H, water, lipid) relaxation and density to visualize musculoskeletal anatomy. Further, nuclear imaging agents, including Na^18^F for positron emission tomography and bisphosphonate tracers for bone scanning, principally target matrix/mineral composition through physio-chemical uptake [3], [4], [5]. Lastly, more recent experimental approaches focused on fluorescent bisphosphonates as static probes for optical detection and validated their potential as structural markers of bone turnover [6], [7], [8].

While imaging approaches focused on measures of morphology have allowed important insights into musculoskeletal pathology, there has been a relative dearth in the development of functional measures of bone biology [9]. Although limited in number, methods for imaging bone biology have demonstrated great potential. For example, one study used a commercially available optical probe for detection of the osteoclast enzyme cathepsin K, a marker of bone resorption [10]. While very promising, use of fluorescence techniques is best suited for superficial applications and does not offer anatomical localization of the light signal. Further, it is unknown if this approach can be accurately quantified in humans. In addition to imaging osteoclasts, osteoblast activity during bone formation has been monitored in transgenic mice expressing a bioluminescence imaging reporter gene under the control of the osteocalcin promoter [11], [12]. However, this strategy is limited to pre-clinical models due to the requirement for transgenic animals. Collectively, existing approaches to imaging bone cell activity are considerably restricted in scope and potential clinical application.

**Figure 1:**
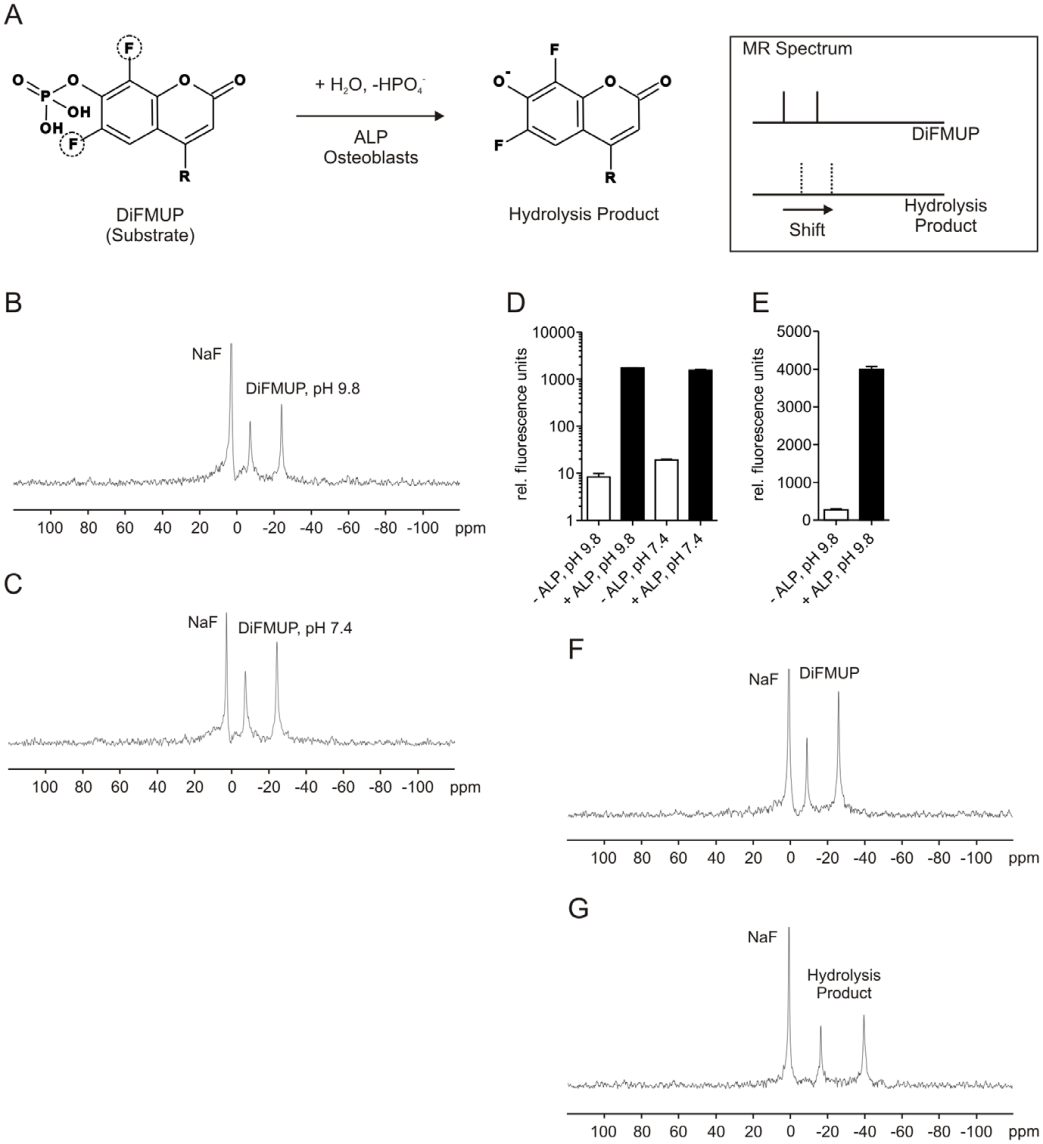
Basic concept and specific detection of the ALP substrate DiFMUP by magnetic resonance spectroscopy. A: In this study, imaging is based on the use of DiFMUP. This small imaging molecule prototype contains two MR-detectable fluorine atoms in direct vicinity to a phosphate group. Prior to hydrolyzation by ALP, DiFMUP is expected to yield a spectrum characteristic of two discrete signals from the fluorine atoms since the molecule is not symmetrical, and hence the fluorine atoms exist in distinct chemical environments. If ALP is present and catalyzes the exchange of the phosphate to a hydroxy group, a significant change in the environment next to the fluorine atoms occurs and is expected to result in a chemical shift variation of the fluorine signals that is detectable by ^19^FMRSI. An added advantage of DiFMUP is the fluorescent property of its hydrolysis product; this allows for the convenient measurement of DiFMUP activation. This feature is not essential for the presented imaging paradigm, however it is used to monitor ALP activity in several subsequent figures. B: The two-peak MR signal of DiFMUP at alkaline pH 9.8 relative to a sodium fluoride (NaF) standard. C: An identical DiFMUP spectrum characteristic was observed under physiological pH 7.4 conditions. D and E: The hydrolysis product of DiFMUP exhibits fluorescence properties, which allows one to record DiFMUP activation. Substantial activation of 50 µM (D) and 5 mM (E) DiFMUP in the presence of ALP was seen in both alkaline and physiological solutions. F and G represent MR spectra of DiFMUP and its hydrolysis product, respectively. The discrete spectrum of the hydrolysis product is seen including resonances from non-equivalent fluorine atoms at −17 ppm and −41 ppm relative to the sodium fluoride reference resonance.

In this contribution, we present a clinically relevant paradigm for the quantitative imaging of bone cell activity. The osteoblast marker alkaline phosphatase (ALP) was visualized directly in bone tissue with anatomical accuracy representing a first step towards the development of imaging osteoblastic cell activity in living subjects, including patients. The tissue non-specific ALP in bone is an extracellular, membrane-bound enzyme that hydrolyzes phosphoric acid monoesters optimally at an alkaline pH [13]. While the *in vivo* substrates of bone ALP remain unclear, ALP is considered an important regulator of mineralization, likely due to its enzymatic degradation of the mineralization inhibitor pyrophosphate [14], [15], [16]. We have selected ALP for proof-of-principle because it is a commonly used clinical marker of bone formation [17]. The imaging paradigm herein is based on the hypothesis that a fluorine-labeled ALP substrate and its hydrolysis product demonstrate MR characteristics amenable to quantitation and visualization using ^19^Fluorine MR spectroscopy (^19^FMRS) and spectroscopic imaging (^19^FMRSI) (Fig. 1A). We demonstrate this concept in a series of *in vitro* and *in situ* experiments.

6,8-difluoro-4-methylumbelliferyl phosphate (DiFMUP) was identified as a prototype small imaging molecule and substrate for ALP. DiFMUP was detected and discerned from its hydrolysis product by ^19^FMRS. Activation of DiFMUP by hydrolysis occurred extracellularly on ALP-expressing osteoblasts. In addition, DiFMUP was activated on purified murine bone tissue in an ALP-dependent fashion and this reaction was quantitatively detected and visualized by ^19^FMRSI.

## Materials and Methods

### Materials

All chemicals, bovine kidney ALP, levamisole (L-tetramisole), and the ALP histochemistry reagents (86-R) were procured from Sigma (St. Louis, MI). The cell lines were obtained from ATCC (Manassas, VA). DiFMUP was purchased from Molecular Probes/Invitrogen (Carlsbad, CA).

### Preparation of DiFMUP and Its Hydrolysis Product

The DiFMUP was dissolved in dimethylsulfoxide (DMSO) and aliquots were stored at −20°C. The final DMSO concentration in all assays was 5% v/v. DiFMUP was assayed in ALP buffer containing 100 mM diethanolamine, pH 9.8, 50 µM MgCl_2_ or in tissue culture medium at physiological pH. Alpha modifed Eagle's medium (α-MEM) supplemented with 10% fetal bovine serum, 2 mM glutamine, and penicillin (100 units/ml)/streptomycin (100 µg/ml) was used because it also served as tissue culture medium for the bone precursor cells studied in this report. Activation of DiFMUP in the presence of 0.1 u purified bovine kidney ALP was measured in a total volume of 500 µl for 1 hr at 37°C.

### Fluorescence Measurements

Only the hydrolysis product of DiFMUP is fluorescent. We selected a 358 nm excitation and 455 nm emission filter set to measure DiFMUP hydrolysis using a Tecan Saphire (San Jose, CA) plate reader with temperature control. We found it convenient to assay samples in 24 well tissue culture plates (BD Falcon, Bedford, MA). Acquisitions were performed in top read mode with a bandwidth of 5 nm or 2.5 nm for 50 µM or 5 mM DiFMUP, respectively. An integration time of 40 µs with no lag time was chosen. The gain was set manually to 100. The z-position was calculated from well A1. Measurements were preceded by a 3 s shake followed by a 3 s rest period and included 100 flashes.

### Measurement of DiFMUP Activation on Bone Precursor Cells

As ALP-positive and negative mouse bone precursor cells, we used 7F2 [18] and MC3T3-E1 #4 [19] cells, respectively. Cells were grown as monolayers on tissue culture plastic in complete α-MEM under a humidified 5% carbon dioxide atmosphere. For ALP histochemistry, cells were plated at 50% or grown to confluence. They were fixed for 10 min in ice-cold phosphate-buffered saline (PBS) containing 10% formalin and subsequently washed with PBS and distilled water. Staining was performed for 20–30 min according to the staining kit's instructions. Then, cells were rinsed in water, counterstained for 1 min in 1 ml Gill's Hematoxylin, washed, and air dried over night. To assay DiFMUP activation on cells, we plated cells in triplicates in Falcon 24 well plates. On average, 16 hrs before the assay 50,000 cells were seeded in a total of 500 µl medium. The enzymatic reaction was initiated by replacing 25 µl medium with 25 µl of 1 mM DiFMUP. Due to an apparent sensitivity of 7F2 cells to DMSO, we maintained the final DMSO concentration at 0.5% v/v.

### Preparation of Bone Tissue

Ethics Statement: Animal studies were performed in accordance with institutional guidelines and the approval of the IACUC at Memorial Sloan-Kettering Cancer Center (Protocol #10-10-020). Bone tissue was harvested post-mortem from 8–12 weeks old Sprague-Dawley rats (Taconic, Hudson, NY). Both tibias were surgically removed, carefully cleaned of soft tissue using a scalpel and stored on ice prior to use. Specimens composed of metaphyseal and diaphyseal bone were separated from bone marrow via at least five grindings in a mortar. Each grinding was carried out in 6 ml ice-cold PBS as 6–7 crushes. After each grinding, the bone marrow-containing supernatant was removed and discarded. The resulting highly purified bone chips had a white-translucent appearance. Fluorescence measurements on bone were carried out in a total volume of 500 µl. Either a single bone chip (3–5 mg) was assayed in ALP buffer (the fluorescence read-out was normalized to the weight of the bone chip) or 15 mg of bone were assessed in medium. Prior to the measurement, samples were kept on ice. The enzymatic reaction was initiated by replacing 25 µl of medium with the same volume of 1 mM DiFMUP immediately before the fluorescence measurement. ^19^FMRS on 30 mg of highly purified bone chips was carried out in a total volume of 450 µl ALP-buffer with 20–25 mM DiFMUP. For imaging, we prepared cortical diaphyseal bone from intact tibia. The bone was split longitudinally into halves approximately 5 mm in length, carefully cleaned from bone marrow, and sandwiched in standard paper towel. Subsequently, we placed the bone core-containing sandwich in a 500 µl cylinder and carefully moisten it with approximately 250 µl of 5 mM DiFMUP in ALP-buffer, added first to the core and then periphery of the sandwich.

### Magnetic Resonance Studies

MR studies were performed on a Bruker (Billerica, MA) Biospec 70/30 small animal imaging system operating at 7 T and equipped with a 200 mT/m gradient coil insert. Experiments were performed with the use of a temperature-control susceptibility-matching water bath [20]. ^19^Fluorine spectra and spectroscopic images were acquired using a custom-built, 1.0-cm inner diameter, two-turn solenoidal coil optimized for transmission and reception at 282 MHz. Spectral data was analyzed using the jMRUI software package [21]. Quantitation of spins was achieved through the use of the AMARES non-linear least squares quantitation algorithm [22]. In accordance with the literature, considerations relevant to data acquired using the external reference technique were included in the analysis. Spin-lattice relaxation times of DiFMUP with rat tibia chips were measured for both the substrate and product and were used to correct for partial saturation effects as described previously [23]. The spin lattice relaxation times for the high and low frequency substrate resonances were measured to be 2.1 s and 1.6 s respectively. The spin lattice relaxation times for the high and low frequency product resonances were measured to be 3.0 s and 2.3 s respectively. Spectroscopic images and parametric maps were generated using Paravision software (Bruker).

### ^19^Fluorine MRS

Samples for ^19^FMRS (n = 5) were contained in 500 µL glass vials (J. G. Finneran Associates, Vineland, NJ) and positioned within the previously described two-turn solenoidal radiofrequency coil. For purposes of quantitation a sealed glass microsphere containing 18 µL of a 150 mM sodium fluoride (NaF) aqueous solution doped with 15 mM Magnevist (Berlex Laboratories Inc., Montville, NJ) was positioned adjacent to the coil. The coil was immersed in the water bath and positioned at the isocenter of the magnet. Subsequent shimming optimized the magnetic field homogeneity to a proton linewidth of approximately 30–60 Hz. Optimized acquisition parameters included a 60° pulse-angle, a 1.7 s pulse repetition time, and a spectral width of 75 kHz in 2048 data points. Spectra were acquired as the average of 353 excitations enabling a temporal resolution of 10 min. per spectrum. Serial spectra of DiFMUP and rat tibia were acquired over a time period of 300 to 600 min following application of DiFMUP. A single, fully-relaxed spectrum of the NaF reference was acquired following the calibration of flip angles to ensure accurate quantitation.

### ^19^Fluorine MRSI

The samples (n = 3) for imaging were prepared for experiments as described above. A chemical shift image sequence was used to acquire a two-dimensional image with thirteen phase encode steps at an in-plane resolution of 1.8×1.8 mm (FOV = 24 mm). Phase encode steps were acquired as the weighted average of 8500 FID signals in 2048 data points with a spectral width of 50 kHz, 60° flip angle and 1.7 s repetition time. The total imaging time was four hours. Prior to MRS imaging, a proton image was acquired at 300 MHz for anatomic localization of the ^19^FMRSI spectra. A rapid acquisition with relaxation enhancement (RARE) sequence was used with 24 mm FOV, 192×256 matrix size, 6 signal averages, 1000 ms repetition time and 102.5 ms echo time. A single, fully-relaxed spectrum of the NaF reference was subsequently following the calibration of flip angles to ensure accurate quantitation.

### Kinetic Modeling of Serial ^19^FMRS Data

The ALP enzyme kinetics were modeled as a simple three-compartment system. The three compartments correspond to: 1) unbound DiFMUP substrate [*S*], 2) the enzyme-substrate complex [*ES*] and 3) the DiFMUP hydrolysis product [*P*] concentrations. All compartments were assumed to be well mixed. The DiFMUP substrate was introduced as a bolus of known concentration and was assumed to bind to the enzyme in a saturable process. The rate of product formation was assumed to be unidirectional and proportional to the enzyme-substrate complex concentration. The unbound enzyme concentration was taken to be the total (bound and unbound) concentration of enzyme, parameterized by *E_0_*, minus [*ES*].

In mathematical terms, this system is described by the following system of equations:
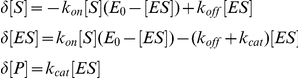
where the initial conditions for [*S*], [*ES*] and [*P*] are known (the latter two being zero and the former being the bolus concentration) and with parameter *k_on_* in units of mM^−1^·min^−1^ and, *k_off_* and *k_cat_* in units of min^−1^, and *E_0_* a scalar parameter in units of mM.

Bound substrate [ES] is not seen by ^19^FMRS, thus the measured substrate concentration directly reflects [S]. The model was used to simultaneously fit the time-course of both [*S*] and [*P*], minimizing the sum of the squares of the residuals while varying the values of *k_on_*, *k_cat_* and [*E*] using a nonlinear search procedure. The system of differential equations was solved and the fitting procedure was performed, using code written in Matlab. Four parameter values, *k_on_*, *k_off_*, *k_cat_* and *E_0_*, were independently determined for each of the three serial time-course experiments. All measured data points were weighted equally during the fitting process.

### Modeling of ^19^FMRSI Data

The model of the FMRSI data made use of the same system of equations applied to serial ^19^FMRS datasets except in this case the parameter values for *k_on_*, *k_off_* and *k_cat_* were fixed at the means of the values determined in the serial experiments. Only *E_0_* was allowed to vary for each image pixel along with a new parameter *w*, which accounted for the partial volume averaging of a given pixel with air or the walls of the vessel. Values for *w* were bound to a maximum value of one and for each pixel a single value of *w* was used to scale both the modeled DiFMUP substrate and hydrolysis product concentrations. The mean values of the modeled substrate and product time-courses over the period of the ^19^FMRSI measurement (60 to 300 min post injection) were fitted to measured concentrations, minimizing the sum of the squared errors while varying *E_0_* for each pixel.

## Results

### MRS Detection of Hydrolysis of DiFMUP In Vitro

To assess the feasibility of DiFMUP detection by ^19^FMRS, the compound was prepared in alkaline (ALP-buffer) or physiological (tissue culture medium) solution. DiFMUP was detected as a two peak spectrum representing non-equivalent fluorine atoms at −10 ppm and −27 ppm relative to the sodium fluoride reference resonance (Fig. 1B). No change in the DiFMUP spectrum was noted between ALP-buffer and medium (Fig. 1B and C). Next, DiFMUP was activated via hydrolysis in the presence of purified ALP. Utilizing the fluorescent properties of the DiFMUP hydrolysis product, a strong increase (100-fold) in fluorescence was registered following a one hour incubation (Fig. 1D). A robust increase (20-fold) in fluorescence was also observed at millimolar concentrations of DiFMUP; however, fluorescence quenching was noted due to a high fluorophore concentration (Fig. 1E). ^19^FMRS was subsequently applied to measure the activation of DiFMUP by purified ALP. As hypothesized, a characteristic four peak spectrum was observed with a pronounced chemical shift of two additional resonances relative to those seen for DiFMUP corresponding to the non-equivalent fluorine atoms of the hydrolysis product. (Fig. 1F and G). Thus, ^19^FMRS was able to discern DiFMUP and its hydrolysis product.

### Extracellular Hydrolysis of DiFMUP in ALP Positive Bone Cells

**Figure 2:**
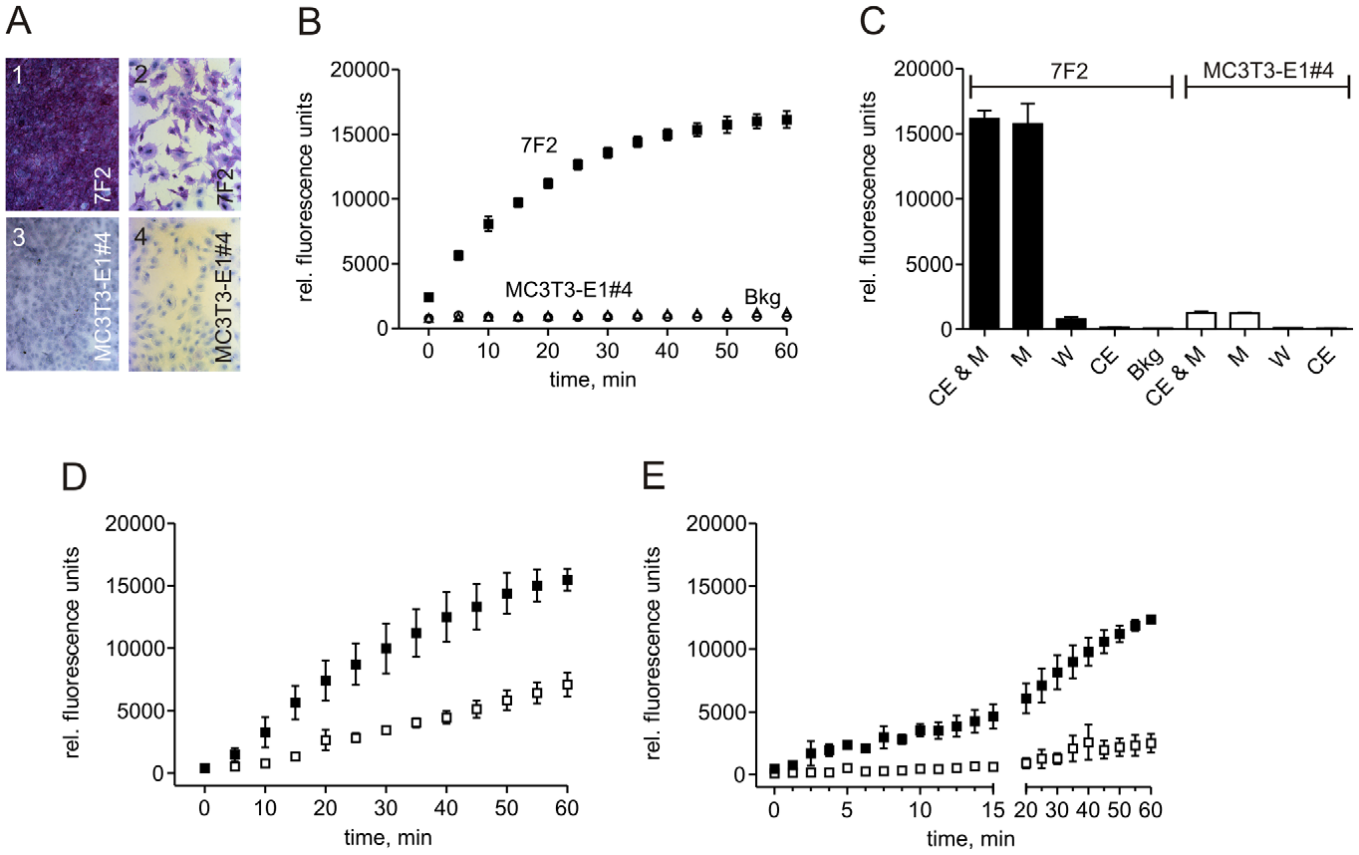
ALP-dependent DiFMUP activation on the surface of ALP-positive osteoblastic bone cells and in the presence of rat tibial bone. A: Histochemistry detected ALP expression as violet-red staining on 7F2 cells as shown in low and high magnification images 1 and 2, respectively. In contrast, no ALP expression was detectable on the MC3T3-E1#4 cells shown also in low (3) and high magnification (4). B: Activation (hydrolysis) of DiFMUP occurred on 7F2 cells (closed squares), but not on ALP-negative MC3T3-E1#4 cells (open triangles). No-cell background (Bkg) measurements are also presented (open circles). C: Following the separation of cells (CE) and medium (M) and a single wash step (W), the vast majority of the hydrolysis product was found in the medium. Mean values and standard deviations are shown (n = 3). D: DiFMUP in physiological solution was activated in a time-dependent fashion in the presence of highly purified rat tibia bone (closed squares). Addition of the ALP inhibitor levamisole reduced DiFMUP activation (open squares). E: DiFMUP in alkaline solution was activated by a single highly purified tibia bone chip (closed squares). Presence of levamisole significantly suppressed the activation (open squares). Mean values and standard deviations are shown (n = 3).

To test the activation of DiFMUP by ALP-expressing osteoblastic bone cells, we compared ALP-positive 7F2 cells (Fig. 2A, panel 1 and 2) to MC3T3-E1#4 cells which were negative for ALP (Fig. 2A, panel 3 and 4). Activation of DiFMUP was restricted to the ALP-expressing 7F2 cells (Fig. 2B) as the fluorescence registered from the MC3T3-E1#4 cells remained at background levels (Fig. 2B). Next, we analyzed the distribution of the hydrolysis product. Approximately 98% of the hydrolysis product was recovered from the medium as compared to the cell monolayer (Fig. 2C). Together, these data demonstrate the extracellular hydrolysis of DiFMUP by ALP-positive osteoblastic bone cells.

### Efficient ALP-dependent Activation of DiFMUP in Bone Tissue

The extracellular activation of DiFMUP by ALP-positive bone cells in tissue culture suggested an ALP-dependent hydrolysis within bone tissue. To investigate this possibility, rat tibia was prepared. Highly purified bone tissue maintained in medium was found to activate DiFMUP (Fig. 2D). The course of DiFMUP activation was comparable to the one observed in the presence of ALP-positive 7F2 cells (Fig. 2B). As shown in Fig. 2D, the hydrolysis was significantly reduced in the presence of 2 mM levamisole, a known ALP inhibitor. A comparable inhibition was seen with 10 mM EDTA (not shown). The accelerated enzymatic reaction in ALP-buffer enabled measurements on single pure bone chips (Fig. 2E). In comparison to the measurement in medium, a more pronounced inhibition by levamisole was seen and resulted in an approximately 80% reduction in fluorescence after 60 min (Fig. 2E).

### Quantitative Imaging of Alkaline Phosphatase in Bone Tissue

**Figure 3:**
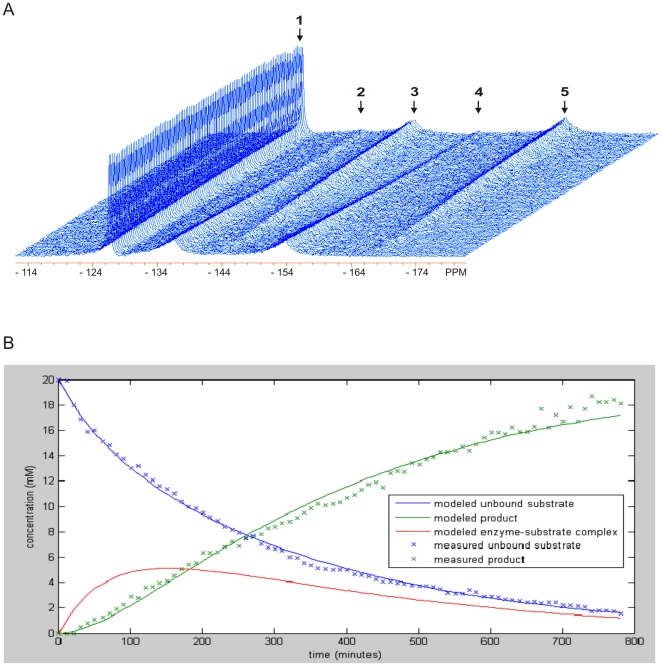
Pharmacokinetics of ALP-dependent DiFMUP activation in the presence of rat tibial bone. A: Representative serial ^19^FMR spectra of DiFMUP and its hydrolysis product in the presence of rat bone. Spectral acquisition was initiated 6 minutes after addition of DiFMUP (20–25 mM range) to highly purified rat bone chips and serial spectra were acquired with 10 min temporal resolution. Peak assignments are as described for previously demonstrated spectra including (1) sodium fluoride, (2,4) DiFMUP and (3,5) DiFMUP hydrolysis product. B: The measured and modeled time course kinetics of unbound DiFMUP (x, —) and its hydrolysis product (x, —) for the ^19^FMR spectra seen in Fig. 3A. The modeled ALP bound DiFMUP (—) is also shown for comparison.

To achieve ^19^FMRSI of ALP activity in bone tissue, we first performed serial ^19^FMRS on highly purified rat cortical bone chips in solution. These serial ^19^FMRS time series measurements delineated the kinetics of ALP-mediated activation of substrate to its hydrolysis product and demonstrated that ^19^FMRS is an effective method for detecting ALP-mediated activation of DiFMUP to its hydrolysis product. (Fig. 3A). Absolute metabolite concentrations derived from these ^19^FMRS time series measurements were fit to a pharmacokinetic model describing the kinetics of bone alkaline phosphatase activity. The result of the fitting procedure for a representative serial experiment is shown in (Fig. 3B). The mean and coefficient of variation of each parameter over the three experiments was calculated to be: *k_on_* 1.72×10^−4^ mM^−1^·min^−1^ (±22%), *k_off_* 2.03×10^−3^ min^−1^ (±61%), *k_cat_* 5.27×10^−3^ min^−1^ (±39%), and *E_0_* 33.6 mM (±31%).

**Figure 4:**
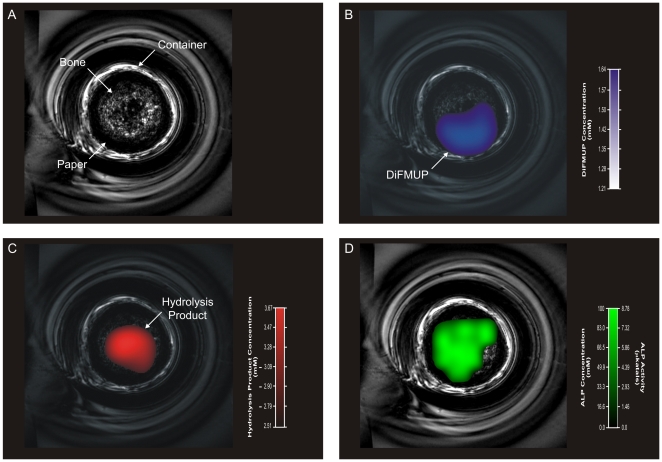
Non-invasive imaging of ALP activity in rat tibia cortical bone. A: RARE ^1^H images of the bone sample anatomy including the rat tibia cortical bone core within the glass vial. B and C: ^19^FMRSI-derived parametric maps of regional DiFMUP and hydrolysis product concentrations overlaid onto RARE ^1^H images of the bone sample (B and C, respectively). D: ^19^FMRSI-derived parametric maps of regional ALP concentration and activity overlaid onto RARE ^1^H images of the bone sample.

This relationship allowed the application of the pharmacokinetic model to achieve quantitative images of bone alkaline phosphatase activity in living bone tissue. We prepared a sample in which a core of bone slivers was wrapped with paper towel soaked in 5 mM DiFMUP. A high resolution RARE MR image was initially obtained for the purpose of voxel prescription and co-registration (Fig. 4A). Two-dimensional ^19^FMRS images (n = 3) were acquired demonstrating the concentrations of DiFMUP substrate and hydrolysis product in a 0.1 cm^3^ volume with an in-plane resolution of 1.8×1.8 mm (Fig. 4B and C, respectively). We observed that the DiFMUP was mostly present in the periphery of the sample (Fig. 4B), while the hydrolysis product localized to the region of ALP enzymatic activity within the bone core (Fig. 4C). The measured concentrations of DiFMUP and its hydrolysis product enabled the generation of parametric maps of regional ALP concentration and activity (Fig. 4D). Together, these data demonstrate imaging of osteogenic bone cell activity. In allowing the quantitative imaging of regional ALP enzymatic activity this approach provides an ideal platform for the further development of quantitative imaging of bone cell activity in living subjects.

## Discussion

Quantitative, diagnostic imaging of the biology which underlies post-natal bone remodeling and repair as well as metabolic bone diseases and skeletal malignancies, has been postulated as important for the development of next-generation clinical musculoskeletal imaging [9], [24], [25], [26], [27], [28], [29]. Toward this goal, we demonstrate proof-of-principle quantitative imaging of bone cell activity in explanted living bone. The activity of the bone cell marker ALP was exploited to enable the direct visualization of bone cells “at work”, i.e. osteoblasts forming bone. Imaging cellular bone markers is a logical translation from their routine use in clinical chemistry where they have been proven to be valuable indicators of bone biology [30]. In contrast to clinical chemistry, however, non-invasive imaging offers striking advantages including the non-destructive measurement of biological activity *in vivo* and in real-time allowing serial measurements within bone anatomy. The importance of imaging bone cell activity is underscored by the finding that controlled changes in the activity of bone cells, such as osteoblasts or osteoclasts, precede changes in bone mineral or matrix composition, thus potentially offering earlier and more precise diagnostic imaging in future clinical applications [9], [31]. We describe a paradigm for imaging osteoblast activity that applies ^19^FMRSI in combination with the small imaging molecule prototype DiFMUP, an ALP substrate.

### The small imaging molecule

Small molecule substrates provide an excellent tool for the detection of enzymes such as ALP in basic research and clinical chemistry. A classic example is the frequently used 5-bromo-4-chloro-3-indolyl-phosphate which hydrolyzes into a reactive intermediate that can further react into a dye. Among the large selection of commercially available ALP substrates are also molecules, including adamantly 1,2 dioxetane phosphate derivatives, which disintegrate upon hydrolysis with the concomitant release of chemiluminescent light. In contrast to these two substrate types, other small molecule substrates maintain their integrity forming hydrolysis products that emit visible or fluorescent light. For example, para-nitrophenylphosphate produces a compound of yellow color while latent precursor substrates such as fluorescein diphosphate or 4-methylumbelliferyl phosphate derivatives, including DiFMUP, form fluorophores. The potential for high-throughput in combination with the high sensitivity offered by probes with optical properties have led to their emergence for *in vitro* applications; however, their suitability for *in vivo* imaging in mineralized tissues is limited mainly because of the restricted depth of light penetration. Thus, we have explored molecular detection traits other than optical properties using an established small molecule substrate. DiFMUP was selected as a prototype small imaging molecule because it harbors two MR-detectable ^19^F atoms in direct vicinity to an ALP cleavage site. The advantages of DiFMUP include its availability to the research community and the fluorescence properties of its hydrolysis product, which make it amenable to *in vitro* characterization assays. Importantly, the development of small imaging molecules as substrates for the non-invasive measurement of ALP activity is not restricted to DiFMUP. The essential motif within the DiFMUP molecule conferring the potential for non-invasive MRSI of bone cell activity is limited to the inclusion of a naturally abundant ^19^Flourine atom and an ALP-cleavage site. As this motif comprises a relatively small number of structural elements, it can potentially be integrated into a large number of compounds in order to generate rationally designed small imaging molecules for bone diagnostics. The disadvantages of the prototype small imaging molecule DiFMUP include a limited specificity and a lack of data supporting *in vivo* use. Given the similarity among the active sites among several tissue non-specific ALP isotypes it may be difficult to achieve high selectivity for ALP in bone. This limitation may be obviated by a local delivery strategy which may include local administration of the small imaging molecule, the use of a bone cell-activated precursor, or the application of bone seeking carriers for targeted delivery.

### Imaging

Magnetic resonance imaging continues to play an increasing role in the clinical diagnosis of musculoskeletal disease. In addition, many institutions are engaged in MR research using dedicated scanners that can accommodate both mice and rats making MRSI an attractive translational imaging method. In comparison to optical imaging of bone [6], [7], [8], [10], [11], [12], MR technology provides unrestricted deep-tissue imaging, unmatched anatomical detail, and absolute quantitation. The limitations of MRI include relatively high costs and labor demand per scan as well as field strength-dependent sensitivity. In previous pre-clinical studies in mice, we were able to detect concentrations as low as 1 mM of fluorinated drugs in tumor tissue at 7 T [32]. The increased sensitivity afforded by the use of higher magnetic field strengths in clinical imaging offers the potential for the increased application of heteronuclear MRSI. The described approach offers the potential to combine functional measures of bone biology using MRSI with the wealth of knowledge that has been accrued using ^1^H MR imaging of musculoskeletal anatomy. To the best of our knowledge, ^19^Fluorine-based MRSI has not previously been applied for the imaging of enzymatic activity in skeletal tissue. The versatility of this paradigm suggests that it can be adapted for additional applications in musculoskeletal biology including activities of enzymes other than ALP, such as osteoclast-associated proteinase activity.

The described imaging strategy may be implemented pre-clinically or clinically on existing MR scanners; however, future applications combining the described MR-based imaging with established nuclear medicine techniques hold the potential to augment the diagnostic information provided by either approach when used as a single modality. For example, while Na^18^F positron emission tomography (PET) permits the sensitive detection of abnormalities in the bone mineral/matrix composition, the predictive diagnostic value of ^18^Fluorine imaging is limited by the inability to distinguish whether areas of mineral/matrix alteration are associated with further bone resorption, new bone formation, or solely represent areas of devitalized bone/matrix. The small imaging molecule-based MRSI reported here will be able to add a layer of biological information to the ^18^Fluorine imaging because it can detect and measure bone formation in areas of altered bone mineral. Thus, the osteogenic potential of such areas can be determined, likely resulting in imaging with increased diagnostic precision and prognostic capability. Another potential application of a combination of MRSI of osteoblast activity and nuclear imaging relates to the diagnosis of primary and secondary skeletal cancers. In these diseases, PET would deliver metabolic tumor imaging, while the small imaging molecule-based MRSI would image the bony environment of the tumor, thereby examining the nature of the cancer-bone interaction. We anticipate that the physical principle that governs the presented imaging is fully compatible with all standard nuclear imaging for musculoskeletal disease including single photon and positron emission techniques. Integration of the latter with MRSI is attractive because both MRSI and PET allow tomographic imaging and ^1^H MRI, which is an integral part of MRSI, can provide high-resolution imaging of the underlying anatomy for both the MRS and PET. The feasibility of MR/PET integrated dual modality imaging is not only supported by recent pre-clinical research [33] but underscored by the development and clinical implementation of MRI/PET scanners [34], [35]. Collectively, potential applications of combined MRSI of osteoblast activity and Na^18^F or ^18^FDG PET include the evaluation metabolic bone disease, infection, as well as primary and secondary skeletal cancers.

### Conclusions

Taken together, we have presented the first example of direct molecular imaging of cell activity associated with bone formation. We have demonstrated the feasibility of anatomically accurate visualization of ALP activity in bone tissue. The MR/small imaging molecule-based paradigm has implications for both preclinical and clinical imaging. On-going preclinical studies are focusing on the added benefits this approach may offer in models of musculoskeletal disease. These early studies will lay the foundation for the development of this clinically relevant approach toward *in vivo* preclinical studies and suggest the potential for applications in patients.

## References

[1] Erickson SJ (1997). High-resolution imaging of the musculoskeletal system.. Radiology.

[2] Augat P, Eckstein F (2008). Quantitative imaging of musculoskeletal tissue.. Annu Rev Biomed Eng.

[3] Jones AG, Francis MD, Davis MA (1976). Bone scanning: radionuclidic reaction mechanisms.. Semin Nucl Med.

[4] Genant HK, Bautovich GJ, Singh M, Lathrop KA, Harper PV (1974). Bone-seeking radionuclides: an in vivo study of factors affecting skeletal uptake.. Radiology.

[5] Kaye M, Silverton S, Rosenthall L (1975). Technetium-99m-pyrophosphate: studies in vivo and in vitro.. J Nucl Med.

[6] Zaheer A, Lenkinski RE, Mahmood A, Jones AG, Cantley LC (2001). In vivo near-infrared fluorescence imaging of osteoblastic activity.. Nat Biotechnol.

[7] Kozloff KM, Weissleder R, Mahmood U (2007). Noninvasive optical detection of bone mineral.. J Bone Miner Res.

[8] Kozloff KM, Volakis LI, Marini JC, Caird MS (2010). Near-infrared fluorescent probe traces bisphosphonate delivery and retention in vivo.. J Bone Miner Res.

[9] Biswal S, Resnick DL, Hoffman JM, Gambhir SS (2007). Molecular imaging: integration of molecular imaging into the musculoskeletal imaging practice.. Radiology.

[10] Kozloff KM, Quinti L, Patntirapong S, Hauschka PV, Tung CH (2009). Non-invasive optical detection of cathepsin K-mediated fluorescence reveals osteoclast activity in vitro and in vivo.. Bone.

[11] Honigman A, Zeira E, Ohana P, Abramovitz R, Tavor E (2001). Imaging transgene expression in live animals.. Mol Ther.

[12] Iris B, Zilberman Y, Zeira E, Galun E, Honigman A (2003). Molecular imaging of the skeleton: quantitative real-time bioluminescence monitoring gene expression in bone repair and development.. J Bone Miner Res.

[13] Kaplan MM (1972). Alkaline phosphatase.. N Engl J Med.

[14] Hessle L, Johnson KA, Anderson HC, Narisawa S, Sali A (2002). Tissue-nonspecific alkaline phosphatase and plasma cell membrane glycoprotein-1 are central antagonistic regulators of bone mineralization.. Proc Natl Acad Sci U S A.

[15] Fedde KN, Blair L, Silverstein J, Coburn SP, Ryan LM (1999). Alkaline phosphatase knock-out mice recapitulate the metabolic and skeletal defects of infantile hypophosphatasia.. J Bone Miner Res.

[16] Whyte MP (1994). Hypophosphatasia and the role of alkaline phosphatase in skeletal mineralization.. Endocr Rev.

[17] Pagani F, Francucci CM, Moro L (2005). Markers of bone turnover: biochemical and clinical perspectives.. J Endocrinol Invest.

[18] Thompson DL, Lum KD, Nygaard SC, Kuestner RE, Kelly KA (1998). The derivation and characterization of stromal cell lines from the bone marrow of p53−/− mice: new insights into osteoblast and adipocyte differentiation.. J Bone Miner Res.

[19] Wang D, Christensen K, Chawla K, Xiao G, Krebsbach PH (1999). Isolation and characterization of MC3T3-E1 preosteoblast subclones with distinct in vitro and in vivo differentiation/mineralization potential.. J Bone Miner Res.

[20] Balloon D, Mahmood U, Jakubowski A, Koutcher JA (1993). Resolution enhanced NMR spectroscopy in biological systems via magnetic susceptibility matched sample immersion chambers.. Magn Reson Med.

[21] Naressi A, Couturier C, Devos JM, Janssen M, Mangeat C (2001). Java-based Graphical User Interface for the MRUI Quantitation Package.. MAGMA.

[22] Vanhamme L, van den Boogaart A, Van Huffel S (1997). Improved method for accurate and efficient quantification of MRS data with use of prior knowledge.. J Magn Reson.

[23] Gade TP, Spees WM, Le HC, Zakian KL, Ponomarev V (2004). In vivo 5-fluorouracil and fluoronucleotide T1 relaxation time measurements using the variable nutation angle method.. Magn Reson Med.

[24] Biswal S (2003). Molecular imaging of musculoskeletal diseases.. Semin Musculoskelet Radiol.

[25] de Boer J, van Blitterswijk C, Lowik C (2006). Bioluminescent imaging: emerging technology for non-invasive imaging of bone tissue engineering.. Biomaterials.

[26] Kaijzel EL, Karperien M, van der Horst G, van der Pluijm G, Lowik CW (2006). Cell-based and molecular imaging tools for validating new therapies in the treatment of bone metabolic disorders and metastases.. Curr Opin Mol Ther.

[27] Mayer-Kuckuk P, Boskey AL (2006). Molecular imaging promotes progress in orthopedic research.. Bone.

[28] Pelled G, Gazit D (2004). Imaging using osteocalcin-luciferase.. J Musculoskelet Neuronal Interact.

[29] Thornton MM (2004). Multi-modality imaging of musculoskeletal disease in small animals.. J Musculoskelet Neuronal Interact.

[30] Seibel MJ, Eastell R, Grundberg CM, Hannon R, Pols HAP, Bilezikian JP, Raisz LG, Rodan GA (2002). Biochemical markers of bone metabolism.. Principles of Bone Biology.

[31] Reumann MK, Weiser MC, Mayer-Kuckuk P (2010). Musculoskeletal molecular imaging: a comprehensive overview.. Trends Biotechnol.

[32] Gade TP, Koutcher JA, Spees WM, Beattie BJ, Ponomarev V (2008). Imaging transgene activity in vivo.. Cancer Res.

[33] Wehrl HF, Judenhofer MS, Wiehr S, Pichler BJ (2009). Pre-clinical PET/MR: technological advances and new perspectives in biomedical research.. Eur J Nucl Med Mol Imaging.

[34] Pichler BJ, Wehrl HF, Kolb A, Judenhofer MS (2008). Positron emission tomography/magnetic resonance imaging: the next generation of multimodality imaging?. Semin Nucl Med.

[35] Sauter AW, Wehrl HF, Kolb A, Judenhofer MS, Pichler BJ (2010). Combined PET/MRI: one step further in multimodality imaging.. Trends Mol Med.

